# The Role of Activity in Synaptic Degeneration in a Protein Misfolding Disease, Prion Disease

**DOI:** 10.1371/journal.pone.0041182

**Published:** 2012-07-16

**Authors:** Matteo Caleo, Laura Restani, Eleonora Vannini, Zuzana Siskova, Hussain Al-Malki, Ruth Morgan, Vincent O'Connor, V. Hugh Perry

**Affiliations:** 1 National Research Council Neuroscience Institute, Pisa, Italy; 2 Scuola Normale Superiore, Pisa, Italy; 3 Centre for Biological Sciences, University of Southampton, Southampton, United Kingdom; University of Nebraska Medical Center, United States of America

## Abstract

In chronic neurodegenerative diseases associated with aggregates of misfolded proteins (such as Alzheimer's, Parkinson's and prion disease), there is an early degeneration of presynaptic terminals prior to the loss of the neuronal somata. Identifying the mechanisms that govern synapse degeneration is of paramount importance, as cognitive decline is strongly correlated with loss of presynaptic terminals in these disorders. However, very little is known about the processes that link the presence of a misfolded protein to the degeneration of synapses. It has been suggested that the process follows a simple linear sequence in which terminals that become dysfunctional are targeted for death, but there is also evidence that high levels of activity can speed up degeneration. To dissect the role of activity in synapse degeneration, we infused the synaptic blocker botulinum neurotoxin A (BoNT/A) into the hippocampus of mice with prion disease and assessed synapse loss at the electron microscopy level. We found that injection of BoNT/A in naïve mice caused a significant enlargement of excitatory presynaptic terminals in the hippocampus, indicating transmission impairment. Long-lasting blockade of activity by BoNT/A caused only minimal synaptic pathology and no significant activation of microglia. In mice with prion disease infused with BoNT/A, rates of synaptic degeneration were indistinguishable from those observed in control diseased mice. We conclude that silencing synaptic activity neither prevents nor enhances the degree of synapse degeneration in prion disease. These results challenge the idea that dysfunction of synaptic terminals dictates their elimination during prion-induced neurodegeneration.

## Introduction

Since the first suggestion that synaptic loss is associated with the degree of cognitive decline in Alzheimer's disease (AD) [1] there has been a growing body of evidence to show that synapse loss is an early component of the neuropathology of chronic neurodegenerative diseases associated with the accumulation of misfolded proteins such as AD, Parkinson's disease, Huntington's disease and prion disease. In some animal models that mimic aspects of the amyloid accumulation in AD there is loss of synapses but not in all models [2], [3]. In humans with amyotrophic lateral sclerosis (ALS) and animal models of the disease there is loss of neuromuscular junction synapses prior to the degeneration of the neuronal cell body [4], [5]. In laboratory models of prion disease there is well documented degeneration of synapses from the hippocampus prior to the degeneration of neuronal cell bodies [6], [7]. Despite the obvious importance of synapse degeneration in these diseases we know remarkably little about the underlying cellular and molecular events by which a misfolded protein or peptide leads to synapse degeneration.

In the ME7 model of prion disease, a tractable laboratory model of chronic neurodegeneration, synapse degeneration in the stratum radiatum of the hippocampus is characterised by the degeneration of the pre-synaptic compartment prior to the loss or degeneration of the post-synaptic dendritic spine. Biochemical analysis has shown there is a loss of synaptic vesicle proteins, cysteine string protein (CSP), VAMP-2, and synapsin, that precedes the reduction of proteins in the post-synaptic compartment [8]. Electron microscopy studies also reveal degeneration of the pre-synaptic compartment prior to the post-synaptic dendritic spine [6], [7]. These changes are readily quantified as the degenerating presynaptic terminals appear electron dense, there is loss of vesicle integrity and the post-synaptic density (PSD) becomes progressively curved as it envelops the degenerating pre-synaptic terminal [7].

The early loss of synaptic vesicle protein CSP is of particular interest since it has been shown that mice lacking this protein are susceptible to a synaptic degeneration phenotype [9]. Furthermore, it is the most active neurons with a high firing rate, GABAergic neurons, in which the synaptic loss is most apparent [10]. There is also evidence that neuronal activity may play a role in the formation of toxic protein aggregates as in cellular models of spinocerebellar ataxia type 3 [11], in the release of amyloid-ß (Aß) peptides [12], [13] and in Aß mediated spine loss in hippocampal slices [14]. Studies on the SOD1 mouse a model of familial amyotrophic lateral sclerosis (ALS) show that it is the fast-fatigueable neuronal population, those with the highest firing rate, that are the first to degenerate [15]. These data suggest that neuronal activity is involved in degeneration in protein misfolding diseases and synapses with high rates of exo/endocytosis may be particularly vulnerable to disease-induced death. On the other hand, as discussed above, it is evident that disruption of synaptic function (“synaptopathies”) represents a major early component of neurodegenerative conditions. In this scenario, synapses with reduced or impaired activity may be selectively targeted for degeneration and removed from the neuronal network during the early stages of the disease. If this view were correct, pharmacological impairment of synaptic transmission should speed up and aggravate synapse degeneration. Altogether, the role of activity in synapse degeneration in diseases associated with accumulation of misfolded protein remains undefined.

In order to understand whether synaptic activity is an important determinant of synapse degeneration we have investigated whether prolonged blockade of synaptic activity impacts on synapse degeneration in prion disease. In previous studies we have shown that the injection of botulinum toxin serotypes A and E (BoNT/A and BoNT/E) into the rodent hippocampus produces a sustained blockade of synaptic transmission via cleavage of the synaptic protein SNAP-25 (synaptosomal-associated protein of 25 kDa). While the effects of BoNT/E are relatively short-lasting (about two weeks, [16], [17], [18]), the action of BoNT/A is much more persistent and lasts for several months [17], [19]. Due to its long-lasting effects, BoNT/A is widely used in the clinical setting for the treatment of pathologies characterized by hyper-excitability of peripheral nerve terminals [20], [21], [22], [23], [24]. We thus injected BoNT/A into the hippocampus of animals at the early stages of the well-defined model of ME7 prion disease and studied how the blockade of synaptic activity affected the extent of synaptic degeneration.

## Materials and Methods

### Ethics statement

All procedures were performed according to the guidelines of the Italian Ministry of Health for care and maintenance of laboratory animals (law 116/92), and in strict compliance with the European Communities Council Directive n. 86/609/EEC. Animal experimentation at the CNR Neuroscience Institute was approved by the Italian Ministry of Health (authorization # 129/2000−A). Specifically, the experiments described in this study were authorized by the Italian Ministry of Health via decree # 185/2009-B, released on November 4, 2009. All surgical procedures were performed under deep anesthesia and all efforts were made to ameliorate suffering of animals.

### Animals and surgery

C57BL/6N mice were bred and group housed in the animal facility of the CNR Neuroscience Institute in Pisa (Italy). When the mice were 11 to 12 weeks old surgery was performed as previously described [25]. Mice were anesthetized by intraperitoneal injection of Avertin (2,2,2-tribromoethanol solution) (20 ml/kg) and mounted in a stereotaxic frame (David Kopf Instruments, Tujunga, CA). In 8 mice, 1 µl injections of a ME7 prion agent-infected brain (ME7-animals) were made bilaterally into the dorsal hippocampus with a 10-µl Hamilton syringe. The suspension was slowly infused and the needle was left in place for 2 minutes before being slowly withdrawn. Mice were placed in a heated recovery chamber and when fully recovered re-housed in groups and checked daily. At 4 weeks after the initiation of disease, 4 mice were injected into the left hippocampus with BoNT/A (0.2 µl of a 1 nM solution) as described below; 4 additional animals received injection of vehicle solution (2% rat serum albumin in PBS). Mice with ME7 prion disease plus vehicle (ME7+vehicle) or with BoNT/A injection (ME7+BoNT/A animals) were killed at 12 weeks after disease initiation and tissues prepared for electron microscopy as described below.

### Botulinum neurotoxin injections

In C57BL/6N mice a stereotaxically guided injection of BoNT/A (0.2 µl of a 1 nM solution) or vehicle (2% rat serum albumin in PBS) was made into the dorsal hippocampus using fine glass micropipettes as previously described [16], [17]. Animals were killed at 2, 4 and 8 weeks after BoNT/A injections and perfused for electron microscopy as described below. Three animals per group (vehicle and BoNT/A) were taken at each time point. A further group of 8 animals were injected in the hippocampus with BoNT/A (n = 4) or vehicle (n = 4) and killed at 8 weeks. Under deep anesthesia, animals were perfused through the heart with freshly prepared 4% paraformaldehyde and the brains processed for immunocytochemistry (see below). In all BoNT/A injected animals, including those previously injected with ME7, recovery was uneventful and no overt behavioural abnormalities were observed.

### Electron microscopy

For electron microscopy mice were perfused with fixative containing 3.4% paraformaldehyde, 1.25% glutaraldehyde, 0.2% picric acid in 0.1 M phosphate buffer (final pH 7.2 to 7.4) immediately after a short perfusion with heparinized saline. Tissue blocks were cut on a vibratome and the area of CA1 pyramidal layer and stratum radiatum was dissected out. Some adjacent vibratome sections through the dorsal hippocampus were collected in PBS and treated with a reducing agent (0.1 M boric acid, pH 8.5) for 10 min before being processed for flee-floating immunostaining for BoNT/A-truncated SNAP-25.

Microdissected areas were postfixed in 1% osmium tetroxide, dehydrated and embedded in TAAB resin as previously described [7]. Semithin (0.5 to 1 µm) sections were stained (1% v/v toluidine blue in 1% w/v borax) and used to guide further cutting of the specimen block into ultra-thin sections (60 to 70 nm). Ultra-thin sections were picked up onto thin bar mesh copper palladium grids and stained in Reynolds lead stain for 5 minutes. Grids were examined using a Hitachi H7000 transmission electron microscope with a MegaView III digital camera (Soft Imaging System) and subsequently processed using Adobe Photoshop software (Adobe Systems Incorporated, San Jose, CA).

### Immunocytochemistry

Tissue blocks from mice eight weeks after BoNT/A injection (n = 4) and their vehicle controls (n = 4) were sectioned at 40 µm on a freezing microtome (Leica). Sections were through the dorsal hippocampus at approximately 2 mm posterior to bregma, comparable to the region studied by electron microscopy. Sections were stained with an antibody that specifically recognizes the BoNT/A-truncated form of SNAP-25 [17], [26], [27]. Briefly, sections were sections were blocked with 10% normal horse serum in PBS containing 0.25% Triton X-100 and then incubated overnight at room temperature with the anti-cleaved SNAP-25 antibody (1∶500 dilution; a kind gift of Dr. Rossetto, University of Padua). On the following day, sections were incubated with Rhodamine Red X-conjugated secondary antibody (1∶400; Jackson ImmunoResearch) for 2 h at room temperature. For Iba-1 immunostaining, sections were blocked in 10% BSA, 0.3% Triton X-100 in PBS for 1.5 hr at room temperature, then incubated with 1∶500 anti-Iba-1 rabbit polyclonal antibody (Wako, Japan), 1% BSA, 0.1% Triton X-100 in PBS, overnight at room temperature. The signal was revealed by incubation in 1∶400 anti-rabbit secondary antibody conjugated to Rhodamine Red-X (Jackson ImmunoResearch, USA), 1% BSA, 0.1% Triton X-100 in PBS, for 2.5 hr at room temperature. Sections were mounted on glass slides using VectaShield mounting medium (Vector Laboratories, USA). Images of cleaved SNAP-25 and Iba-1 immunoreactivity in the hippocampus were acquired with a confocal laser scanning microscope (Leica Microsystems, Germany).

### Quantitative analysis

Electron microscopy images were taken from the stratum radiatum at a magnification of x20,000 or x40,000. The images were sampled in a quasi-random fashion as previously described [7] but avoiding blood vessels, the major dendrites and rare cell somas within the stratum radiatum. Measurements of the PSD and pre-synaptic terminal areas were performed on asymmetric synapses at 2, 4 and 8 weeks after injection with BoNT/A (or vehicle) on images taken at x20,000 magnification. The area of a PSD was measured only if synaptic vesicles were present in the same section and typical membrane apposition between pre-synaptic and post-synaptic element was present. A total of 115–636 PSDs and pre-synaptic terminals were measured for each time point from three to six different sections at least ten sections apart for both BoNT/A and vehicle injected animals. The areas of pre-synaptic terminals containing synaptic vesicles and PSDs were included only if synaptic terminal profiles were clearly visible. In a subset of the fields taken at x40,000 the size (equivalent diameter), circularity and density of synaptic vesicles was measured in asymmetric synapses from BoNT/A-treated and control animals at 4 weeks after injection. Density of vesicles was measured in 30 synaptic terminals per group from 3 vehicle- and 3 BoNT/A-injected mice. Circularity and vesicle diameter were determined from 1962 and 927 synaptic vesicles from BoNT/A- and vehicle-treated terminals. Measurement of PSD areas, presynaptic terminal areas, vesicle area and circularity were performed using ImageJ software (U.S. National Institutes of Health, http://rsb.info.nih.gov/ij/download.html). The identities of images were coded and only revealed to the observer after the data analysis was complete.

To examine synaptic degeneration, images from prion animals were taken from the stratum radiatum at a magnification of x20,000. The analysis was conducted in ME7-only animals (10, 12, 16 and 18 weeks after injection; n = 3 per time point) and ME7 mice treated with vehicle (ME7+vehicle; n = 4) or BoNT/A (ME7+BoNT/A; n = 4). For each image (52–136 images per condition) we determined the number of degenerating and healthy synapses. We scored as degenerating terminals those boutons with a typical dark appearance and a curved PSD [7]. Results were expressed either as the percentage of degenerating/healthy synapses in each condition or as the mean number of degenerating/healthy synapses per image.

The density of Iba-1-positive cells was quantified by means of the Stereo Investigator software (MicroBrightField, USA), using three-dimensional counting boxes (100 µm×100 µm×30 µm) positioned in the stratum radiatum of the CA1 region. For each animal, at least five sections were examined with the operator blind to experimental treatment.

### Statistics

Statistical analysis was performed with SigmaPlot (version 11). Differences between two groups were assessed with Student's t-test for data normally distributed, and with Mann-Whitney rank sum test for data non-normally distributed. Differences between four groups were evaluated with one way analysis of variance (ANOVA) followed by Holm-Sidak test or Dunn's test. Normality of distributions was assessed with a Kolmogorov-Smirnov test.

## Results

### Effect of BoNT/A on synaptic morphology in naïve mice

The long-term effects of BoNT/A on synapses of the CNS following direct injection into the brain parenchyma have not been previously studied, although there have been reports on the impact of BoNT/A and tetanus neurotoxin on neurons of the peripheral nervous system [28], [29]. We addressed this issue by injecting adult mice into the left hippocampus with BoNT/A (1 nM solution, 0.2 µl; n = 13 animals) or vehicle solution (0.2 µl of 2% rat serum albumin in PBS; n = 13). Animals were killed 2, 4 and 8 weeks after injection and the CA1 stratum radiatum of the treated side was processed for electron microscopy analysis.

**Figure 1:**
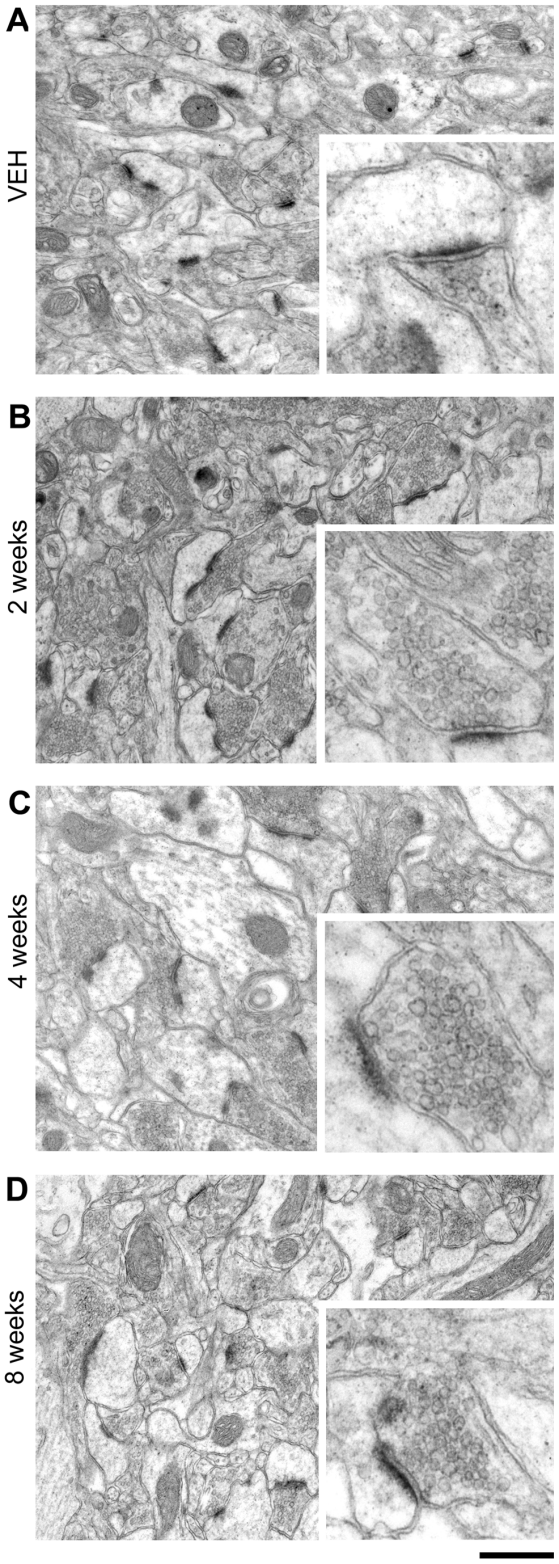
Ultrastructure of BoNT/A-treated hippocampal synapses. Representative electron micrographs of CA1 stratum radiatum in animals treated with vehicle (VEH) and at different times (2, 4 and 8 weeks) after BoNT/A infusion. There was no significant difference between the control groups at 2, 4 and 8 weeks after vehicle injection and the data from these groups has been merged. High magnification pictures are shown in the insets. Note enlarged presynaptic terminals following BoNT/A. Scale bar = 500 nm (250 nm for insets).

**Figure 2:**
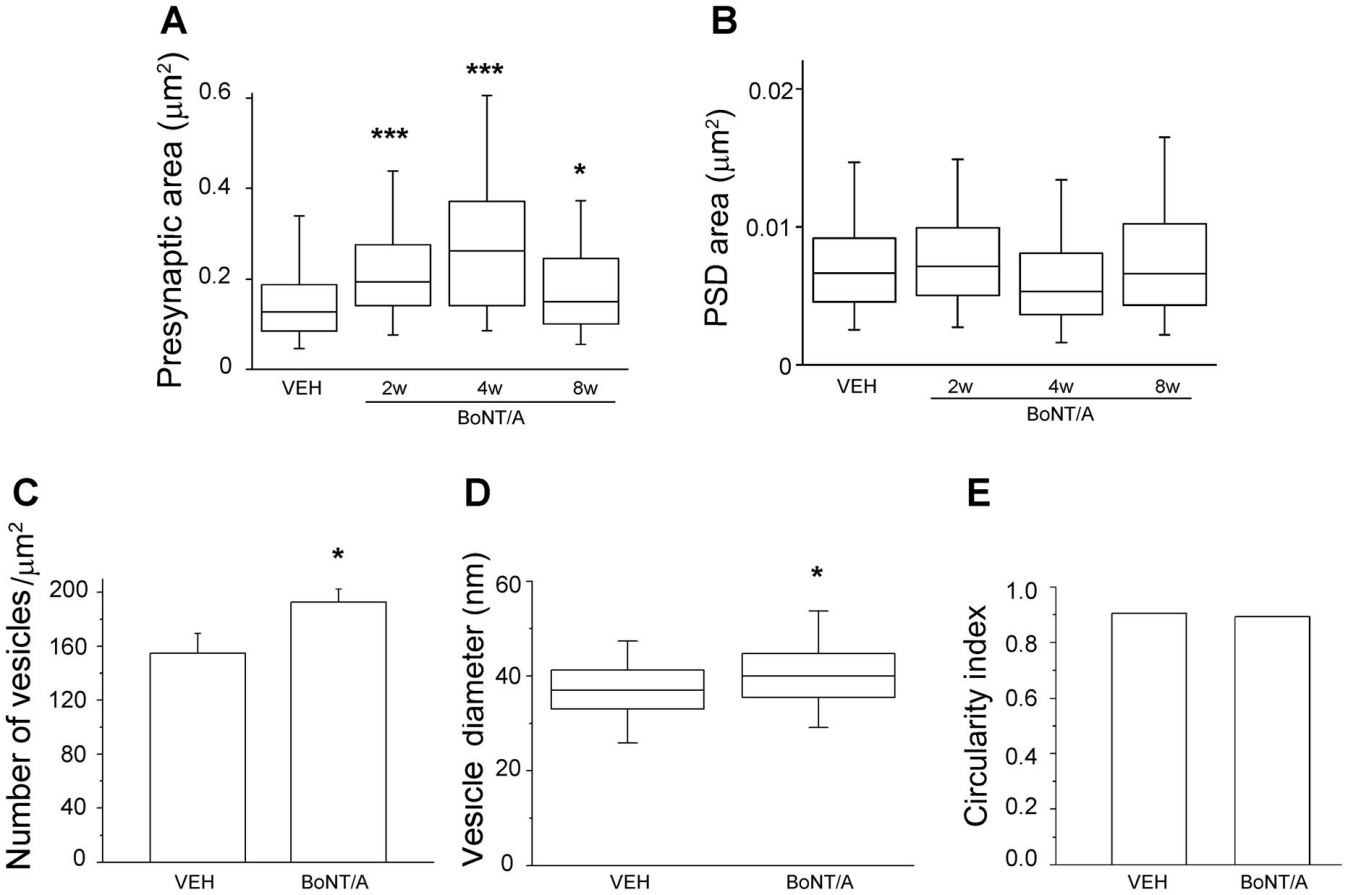
Quantification of the ultrastructural changes in BoNT/A-treated synapses. (A, B) Areas of presynaptic terminals (A) and PSDs (B) in mice infused with vehicle (VEH) or BoNT/A. BoNT/A treatment increases the surface area of presynaptic boutons at all time points examined (2,4 and 8 weeks; one way ANOVA on ranks followed by Dunn's test, p<0.05). PSD areas are unaffected (one way ANOVA on ranks, p>0.05). Data for each group are summarized by a box chart, in which the horizontal lines denote the 25th, 50th, and 75th percentile values, and the error bars denote the 5th and 95th percentile values. Numbers of synapses analyzed are as follows: VEH, n = 636; BoNT/A 2 weeks, n = 505; BoNT/A 4 weeks, n = 185; BoNT/A 8 weeks, n = 195. (C) Density of synaptic vesicles is significantly increased in BoNT/A animals at 4 weeks as compared to vehicle controls (VEH; Student's t-test, p<0.05). Data are mean ± SE. (D) Synaptic vesicle diameter is slightly larger 4 weeks after BoNT/A (Mann-Whitney rank sum test, p<0.05). (E) Circularity of vesicles is identical in vehicle- and BoNT/A-treated terminals (t-test, p>0.05). The SE is too small to be visible. ***, p<0.001; *, p<0.05. Density, diameter and circularity were measured in n = 1959 and n = 926 vesicles, respectively, from BoNT/A and vehicle terminals at 4 weeks.

The most striking feature of the BoNT/A-treated tissue was the presence of enlarged asymmetric pre-synaptic terminals, which were particularly conspicuous at 4 weeks post-injection (Figure 1). No ultrastructural changes were evident in symmetric, presumably GABA-ergic synapses. Apart from the enlarged pre-synaptic compartment, with an apparent increase in the number of vesicles, the pre-synaptic element appeared normal with no increase in lysosomes or other organelles: similarly the post-synaptic densities (PSD) and dendritic spines appeared morphologically normal (Figure 1). We measured the area of the pre- and post-synaptic elements in BoNT/A- and vehicle-injected hippocampi. As shown in Figure 2A the area of the pre-synaptic compartment is larger in BoNT/A animals than controls at all time points (Kruskal-Wallis One Way Analysis of Variance on Ranks, p<0.001; post hoc Dunn's test, p<0.05) with the maximum change present at 4 weeks post injection, where there is a more that two-fold increase relative to controls (p<0.001). The pre-synaptic elements at the 8 week timepoint are then reduced relative to size at 4 weeks and closer to the size seen in vehicle-treated animals (Figure 1A and Figure 2A). In contrast to the pre-synaptic elements the area of PSD is not significantly different from controls at any timepoint examined (Kruskal-Wallis One Way Analysis of Variance on Ranks, p>0.05) (Figure 2B).

Since the pre-synaptic compartment was significantly enlarged we were interested to learn whether there was an increase in the density of the vesicles and if so whether this might reflect the transport of immature vesicles into the pre-synaptic compartment. In the pre-synaptic terminals seen at 4 weeks post injection there is an increase in the density of vesicles (Figure 2C; Student's t-test, p<0.05) consistent with the observation that the area of these synapses is overtly larger and populated with a larger number of vesicles. However, the vesicles of these enlarged synapses are very similar in both size and circularity to the vesicles of naïve synapses (Figure 2D, E; circularity index, t-test, p>0.05). There is a small but significant difference in the size of BoNT/A and control synaptic vesicles (Mann-Whitney Rank Sum test, p<0.05) but there was no evidence of a population of immature vesicles with a more heterogeneous size profile such as we might expect if a population of immature vesicles were being transported from the cell body or formed locally [30]. We did not observe any increase in dense core vesicles, also associated with synapse development, in the BoNT/A treated animals (data not shown).

**Figure 3:**
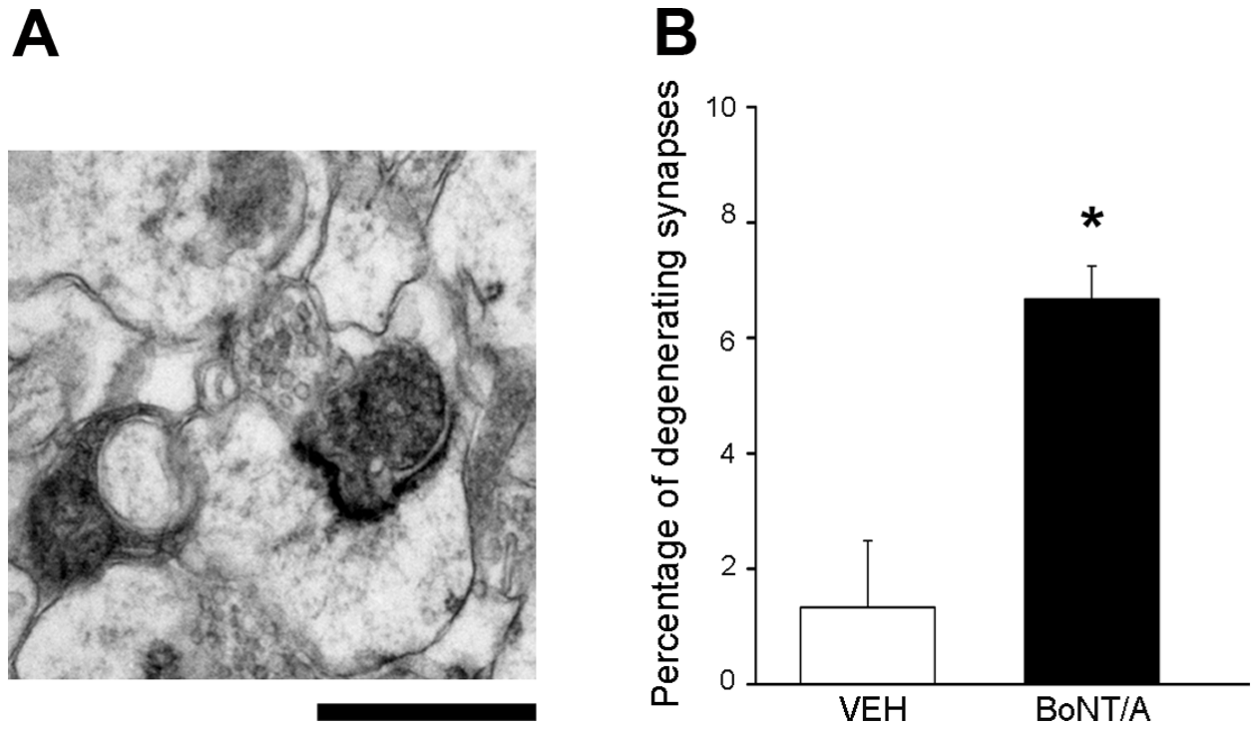
Neuropathological changes in a subset of BoNT/A-treated terminals at 4 **weeks.** (A) Representative electron micrograph showing a degenerating, dark synaptic terminal with curved PSD. Scale bar  = 250 nm. (B) Percentage of degenerating synapses in vehicle (VEH)- and BoNT/A-treated animals. Dark terminals with a curved PSD (<120 deg) were scored as degenerating synapses. *, p<0.05. A total of 100 terminals were scored for each of three vehicle- and BoNT/A-injected animals at 4 weeks.

There was no evidence of neuronal/synaptic degeneration in the EM sections analyzed 2 or 8 weeks after BoNT/A injection. However, in the animals sacrificed at 4 weeks, we detected a subset of dark, electron-dense synapses with “curved” PSDs, that resemble degenerating terminals seen in early stages of prion disease (Fig. 3A). These degenerating terminals represented 6.6% of the total synaptic pool at 4 weeks (Fig. 3B). Since no dark profiles were observed either 2 or 8 weeks after BoNT/A, we interpret this finding as a transient wave of synaptic degeneration caused by activity blockade in a small subset of hippocampal terminals.

**Figure 4:**
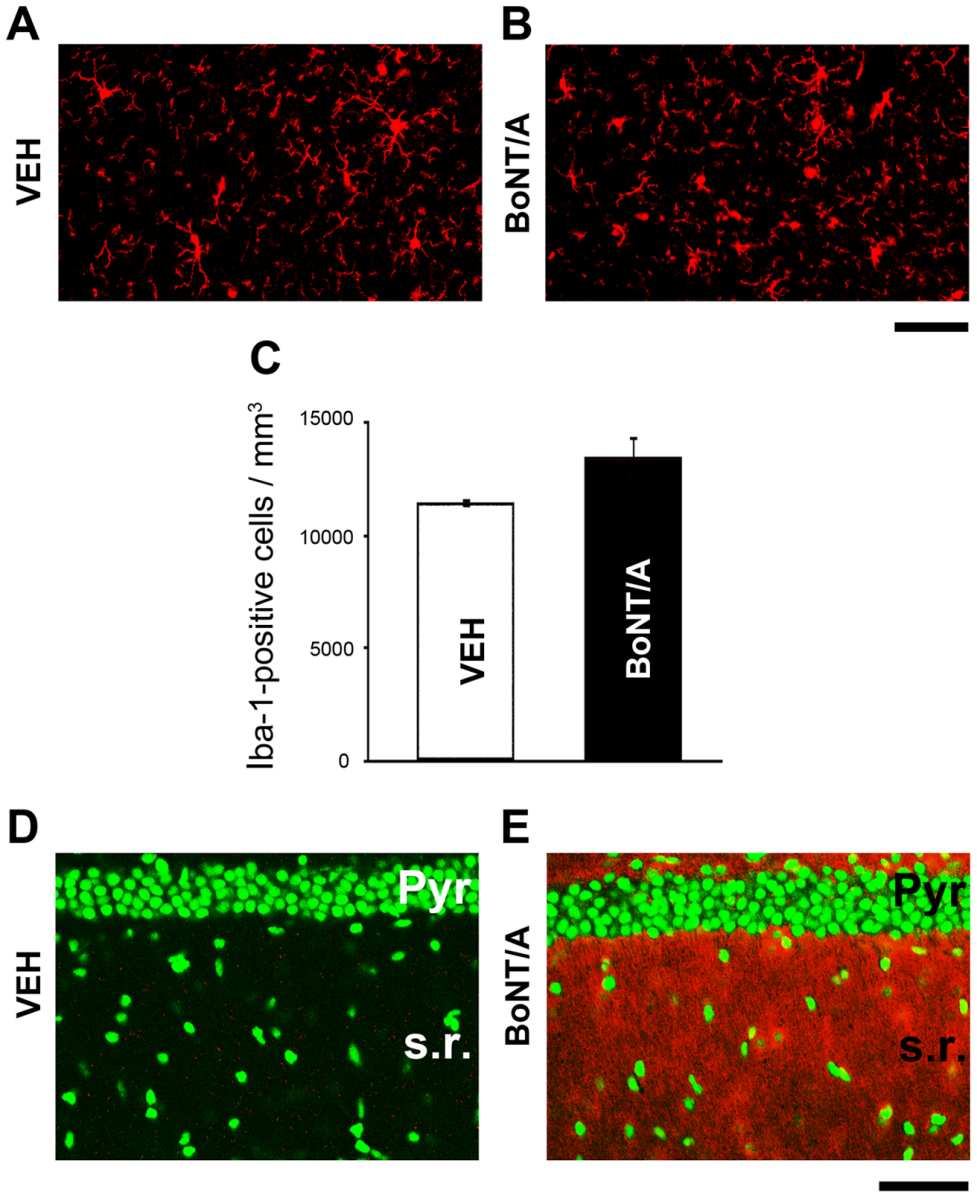
Lack of significant microglial activation 8 weeks after BoNT/A. (A, B) Representative immunostaining for the microglial marker Iba-1 in the CA1 stratum radiatum of mice treated with vehicle (A, VEH) and BoNT/A (B). Scale bar  = 100 µm. (C) Cell countings reveal no significant differences in microglial density between vehicle and BoNT/A-infused animals (t-test, p>0.05). (D, E) Immunostaining for cleaved SNAP-25 (red) demonstrates strong labeling in the CA1 stratum radiatum of BoNT/A-treated animals (E). No labeling is evident in vehicle mice (D). Green, Yoyo-1 nuclear counterstaining. Pyr, stratum pyramidale; s.r., stratum radiatum. Scale bar  = 100 µm.

A sensitive indicator of any pathology in the brain are changes in the morphology and number of microglia [31], [32]. To determine whether there is ongoing synaptic pathology after long-term activity blockade and an associated microglia response, we stained sections from BoNT/A and vehicle animals with Iba-1 to reveal the microglia population 8 weeks after treatment (Figure 4A, B). The microglia in the hippocampus of BoNT/A and naïve animals were similar in morphology. There was a tendency for increased microglial cell density in BoNT/A mice, but this trend was not significant (Figure 4C; t-test, p = 0.12), consistent with the lack of signs of pathology in the electron microscopy sections at 8 weeks. Staining for CD68, a marker of activated microglia, also revealed no significant differences between vehicle and BoNT/A animals at 8 weeks (data not shown). Furthermore, in the electron microscopy there was no evidence of synaptic envelopment or interposition of processes between the pre-and post-synaptic specializations that might be indicative of a microglia response to the silent synapses. Adjacent sections were stained for BoNT/A-truncated SNAP-25 to demonstrate that at 8 weeks post-injection the effect of the toxin was still present (Figure 4D, E). The presence of cleaved SNAP-25 was widespread throughout the dorsal hippocampus, with strong staining in the stratum radiatum (Figure 4D, E) although it should be noted that this does not distinguish between the persistence of the toxin, the delayed turnover of the cleaved fragment or possibly both [17], [27].

### Effect of BoNT/A on synaptic degeneration in prion disease

The features of degeneration of asymmetric synapses in the hippocampus during the progression of ME7 induced prion disease have been previously described [6], [7]. The synaptic vesicles in the pre-synaptic terminal lose their definition and ultrastructural integrity, the pre-synaptic compartment is electron dense, and strikingly the PSD is closely apposed to and appears progressively curved around the degenerating pre-synaptic terminal. The darker and more degenerate the pre-synaptic terminal the more curved the PSD appears [7].

**Figure 5:**
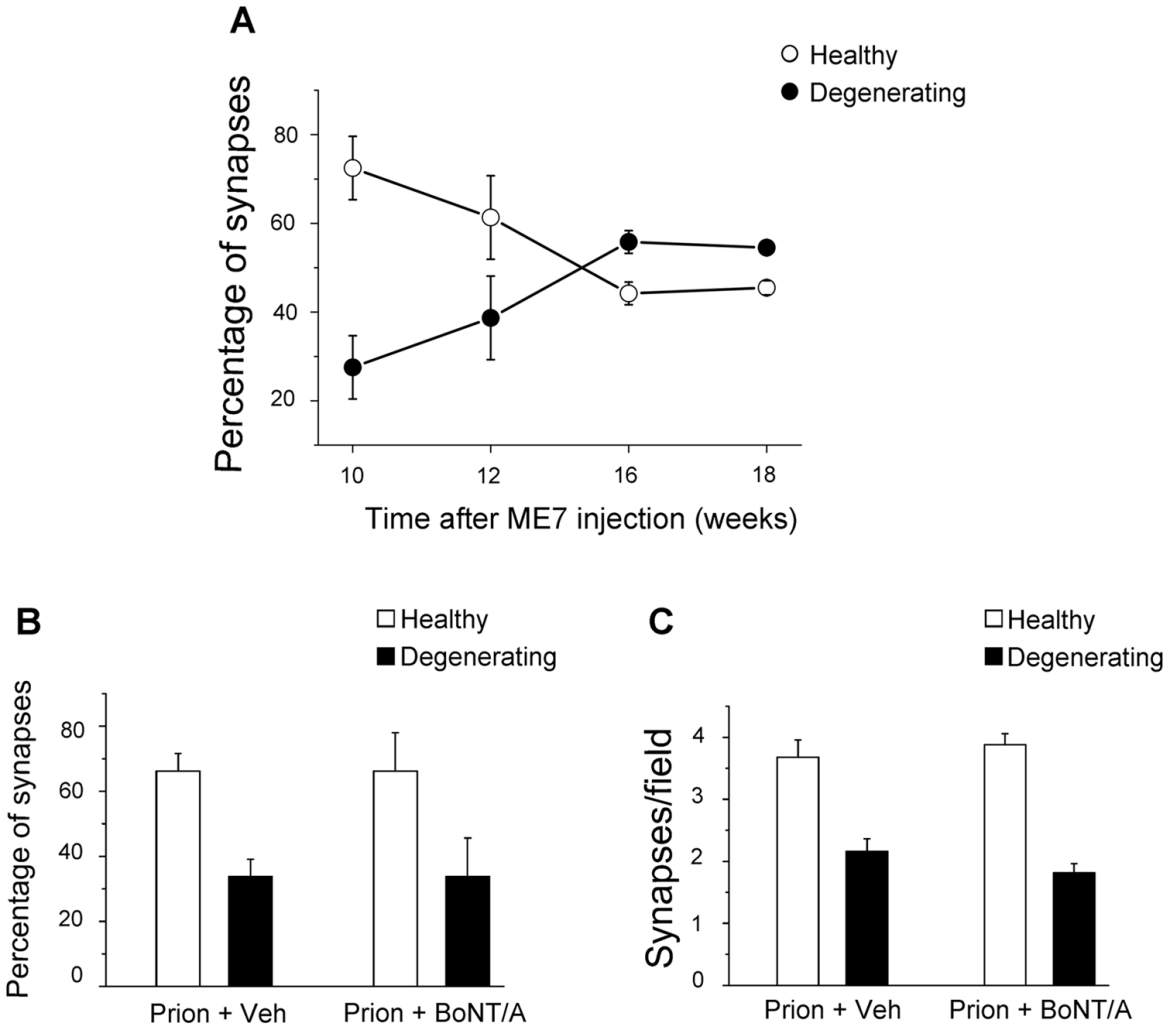
BoNT/A has no significant impact on synaptic degeneration. (A) Percentages of healthy (open circles) and degenerating synapses (filled circles) in the CA1 stratum radiatum during prion disease progression. Degenerating terminals increase while normal boutons decrease in frequency from 10 to 16–18 weeks after delivery of the ME7 agent (one way ANOVA followed by Holm-Sidak test, p<0.05). Numbers of terminals analyzed are as follows: 10 weeks, n = 263; 12 weeks, n = 371; 16 weeks, n = 367; 18 weeks, n = 398. (B) Percentages of healthy (open bars) and degenerating synapses (filled bars) in prion+vehicle (prion+Veh) and prion+BoNT/A mice at 12 weeks into the disease. Percentages are identical in the two groups (t-test, p = 0.97). (C) Density of normal synapses (open bars) and degenerating boutons (filled bars) in prion+vehicle (prion+Veh) and prion+BoNT/A mice at 12 weeks into the disease. There are no statistically significant differences between the two groups (t-test, p>0.19). Number of terminals analyzed are as follows: prion+vehicle, n = 327; prion+BoNT/A, n = 723.

Here, we first determined the time-course of synaptic loss by scoring healthy and degenerating boutons in the CA1 stratum radiatum at different times after intrahippocampal delivery of the ME7 prion agent. Sections were taken from ME7 mice of a previous study [7]. We found that on average about 30% of the boutons were degenerating at 10 weeks. As the disease progresses, the ratio of degenerating synapses increased to reach a maximum 16–18 weeks after ME7 (Fig. 5A). In parallel, the proportion of healthy terminals decreased steadily from 10 to 18 weeks (Fig. 5A). On the basis of these findings, we selected the 12 weeks time-point for the analysis of the effects of synaptic blockade on synapse degeneration. Indeed, this time-point corresponds to an intermediate phase of the degeneration process that allows us to measure BoNT/A-mediated decreases or increases in the degree of synaptic loss.

We have previously shown that immediately after the injection of ME7-infected or normal brain homogenate into the mouse hippocampus there is an acute inflammatory response to the delivery of a high concentration of protein but this resolves by 4 weeks [33]. We thus injected ME7-animals with BoNT/A (n = 4) or vehicle (n = 4) into the dorsal hippocampus at 4 weeks after the initiation of the disease. All animals were killed 12 weeks after ME7. Since the BoNT/A serotype has very long lasting effects [17], [27] we reasoned that silencing synaptic activity for many weeks prior to the onset of significant synaptic degeneration rather than acutely would provide the most likely scenario for an impact on synapse survival.

**Figure 6:**
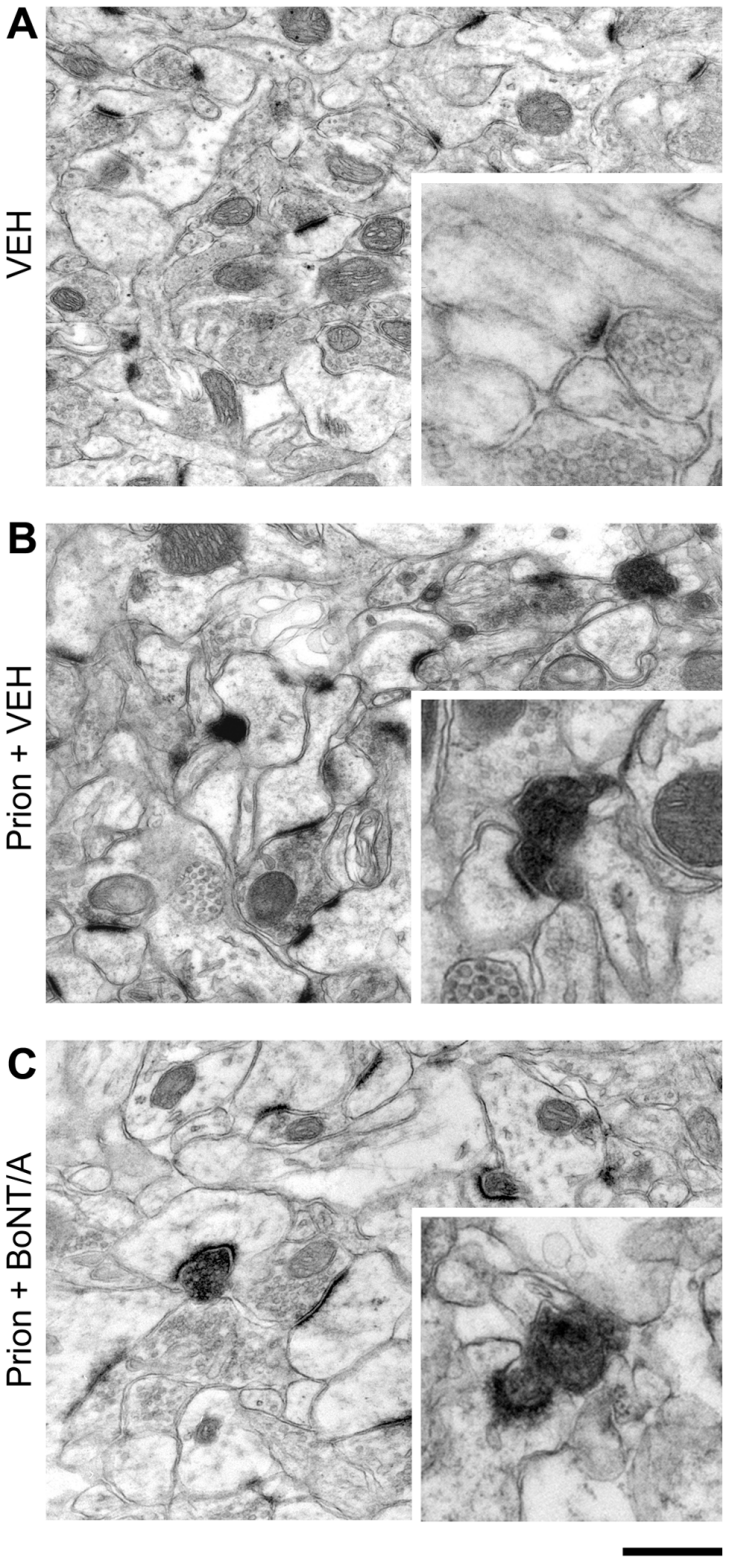
Ultrastructural hallmarks of prion disease are not impacted by BoNT/A treatment. Electron micrographs of CA1 stratum radiatum of the hippocampus illustrating control, vehicle-exposed synapses (A, VEH) and neuropathological changes in prion+vehicle (prion + VEH; B) and prion+BoNT/A animals (C) at 12 weeks following delivery of the ME7 agent. High magnification pictures are shown in the insets. Note degenerating boutons with curved PSDs in both prion+vehicle and prion+BoNT/A samples. Scale bar  = 500 nm (250 nm for insets).

We found that at 12 weeks after the initiation of disease the ratio of degenerating to intact synapses was the same (Student's t-test, p = 0.67) in both the ME7 animals from Siskova et al. (2009) and the ME7+vehicle animals used in this study (Fig. 5A, B). In ME7+BoNT/A animals it was apparent that there were asymmetric synapses undergoing degeneration with the same features as those seen in ME7+vehicle animals, with loss of vesicle integrity, electron dense pre-synaptic compartment and curvature of the PSD around the degenerating pre-synaptic component (Fig. 6). The post-synaptic dendritic spine showed no signs of degeneration (Fig. 6). Quantification of the ratio of degenerating synapses to total synapses revealed no significant differences between ME7+vehicle animals (33.7±5.4%) and ME7+BoNT/A mice (33.8±11.5%; Student's t-test, p = 0.97; Fig. 5B). We also analyzed the absolute density of intact/degenerating synapses per image and again we found that the values of ME7+vehicle and ME7+BoNT/A mice were perfectly superimposable (Student's t-test, p = 0.54 for healthy synapses and p = 0.19 for degenerating boutons; Fig. 5C). To control whether BoNT/A was effective in prion-injected hippocampi, a subset of vibratome sections adjacent to those used for electron microscopy were stained for BoNT/A-truncated SNAP-25 and showed the expected labeling in the stratum radiatum (data not shown). These data demonstrate that BoNT/A-mediated silencing of synaptic activity has no effect on synaptic degeneration.

## Discussion

In this study we have investigated how synaptic activity influences synapse degeneration in mouse prion disease. We hypothesized that silencing synaptic activity would delay or prevent the onset of synapse degeneration since there is evidence to suggest that higher levels of neuronal activity are associated with synapse and neuronal degeneration [10], [15]. To interfere with synaptic activity we employed the clostridial neurotoxin BoNT/A, a bacterial enzyme that causes a persistent and highly selective effect on transmitter release via cleavage of the SNARE protein SNAP-25 [17], [27], [34]. BoNT/A treatment in naïve animals led to the generation of abnormally large asymmetric pre-synaptic terminals in CA1 stratum radiatum, with a greater number of normal appearing vesicles. Prolonged silencing of activity caused only minimal synaptic pathology at four weeks after BoNT/A injection but not at longer time points. When BoNT/A was infused into the prion-diseased mouse hippocampus, rates of synaptic degeneration remained unaltered. Thus, contrary to our expectations, silencing synapse activity neither prevented nor indeed enhanced the degree of asymmetric synapse degeneration in prion disease. We discuss in turn the action of BoNT/A on naïve central synapses and the lack of BoNT/A effects in prion disease.

### BoNT/A in the normal CNS

There are no electron microscopy studies on the long-term consequences of delivering BoNT/A directly to the CNS. Previous electron microscopy studies on the effect of BoNT/A and tetanus neurotoxin on peripheral nerve terminals show the accumulation of synaptic vesicles at the presynaptic site [28], [29]. In this study we have shown that 1 nM BoNT/A, a dose previously shown to abolish excitatory neurotransmission and cell firing in the hippocampus [17], [35], leads to widespread cleavage of SNAP-25 that persists for at least 8 weeks the latest time point examined. We detected ultrastructural changes in asymmetric terminals in the stratum radiatum, the vast majority of which arise from axons of CA3 pyramidal neurons. Following BoNT/A treatment, CA1 terminals were not only enlarged but had a larger complement of synaptic vesicles. These synaptic vesicles had a normal shape and size, and are suggestive of a continuing supply of mature vesicles arriving at synapses and unable to fuse with the plasma membrane to release their neurotransmitter. There was no evidence of immature pleiomorphic vesicles [30] at the pre-synaptic terminal. Presynaptic terminal areas were maximally increased 4 weeks after BoNT/A, with a significant relaxation at 8 weeks. The return of the presynaptic boutons towards their normal size at 8 weeks may reflect an adaptation of the neuron to the lack of activity, as there was clear evidence of persistence of high levels of cleaved SNAP-25 in the hippocampus at 8 weeks (present results and [17]). Of interest is that while the pre-synaptic terminals were enlarged, the PSDs did not show a compensatory change in the absence of neurotransmitter release. It is not the case that PSDs can not enlarge since this is seen during progression of prion disease and interpreted as a reactive response to the loss of synapses in the stratum radiatum [36].

The increase in the density of synaptic vesicles in BoNT/A-treated terminals is consistent with previous ultrastructural work on peripheral neurons incubated with clostridial toxins [28], [29], [34]. Recently, Wree and collaborators reported axonal swellings and varicosities following injection of BoNT/A into the rat striatum. These swellings might correspond to enlarged synaptic terminals as they contained the biosynthetic enzymes for acetylcholine and dopamine [37].

In addition to enlargement of terminals, BoNT/A caused the appearance of a small percentage of dark, degenerating synapses 4 weeks after injection. Since these dark profiles were not detected at other times (2 and 8 weeks), we interpret this finding as a transient wave of degeneration triggered by the activity blockade in a subset of sensitive terminals. It is interesting to note that dark synapses have been seen in association with hypoglossal neurons following injection of BoNT/A into the tongue [38]. Recent evidence showing that BoNT/A may be transferred transneuronally [17], [27] suggests that the toxin may access the presynaptic neuron and cause degeneration in some synapses innervating the hypoglossal motor neurons.

In the long-term (8 weeks), BoNT/A caused no synaptic degeneration and, consistently, there was the absence of a significant microglia response as shown by normal microglial cell densities. It is interesting that the microglia do not respond to the widespread silencing of synaptic activity in the CNS since recent studies have suggested that microglia monitor synaptic health and activity [39], [40]. However, we cannot exclude that more subtle changes in microglia morphology and/or function (not detectable with Iba-1 labeling) occur as a result of synaptic silencing.

### Activity and synaptic degeneration

The degeneration of asymmetric terminals is a conspicuous feature of the early pathology in the ME7 model of prion disease and precedes any detectable degeneration of cell bodies of CA3 or CA1 pyramidal neurons [7], [8]. The precise mechanisms leading to synapse degeneration are poorly understood but as discussed elsewhere [8] do not appear to be simply related to the accumulation of protease resistant prion protein (PrP^Sc^). There is growing body or evidence to show in prion disease that the accumulation of PrP^Sc^ is neither necessary or sufficient to lead to neuronal degeneration [41], [42], but by analogy with studies in Alzheimer's disease soluble oligomeric species of misfolded protein may be critical [43], [44]. How the misfolded protein targets the pre-synaptic terminal leading to degeneration is unclear but early changes in the synaptic vesicle proteins [8] suggest that uptake of the misfolded peptide during the exocytosis-endocytosis cycle may be involved. In keeping with this idea, elevated neural activity has been shown to enhance synapse vulnerability in a mouse model of synaptic degeneration [10]. In Alzheimer's patients, brain areas that develop the most Aß plaques also have the highest resting activity [45]. We thus hypothesized that blockade of synaptic activity may arrest or delay synapse loss during chronic neurodegeneration.

To test this idea, we employed the ME7 mouse model of prion disease that has been previously characterized in detail [46]. The advantage of this model is that the neuropathology can be spatially and temporally controlled by intracerebral delivery of the prion agent. Injection of the ME7 agent into the hippocampus triggers a transient inflammatory response that resolves by 4 weeks [33], and synapse loss with associated behavioural impairments is detected in the stratum radiatum by 12 weeks [47]. Synaptic degeneration increases between 10 and 12 weeks after ME7 injection and reaches a plateau at 16–18 weeks (see Figure 5). We thus chose to infuse BoNT/A in the prion-injected hippocampus at 4 weeks, many weeks prior to first significant loss of synapses but after the first inflammatory phase, and assessed numbers of healthy/degenerating synapses in the CA1 stratum radiatum at 12 weeks into the disease. Thus, synaptic exocytosis was blocked early in disease evolution and the evidence indicates, from staining for cleaved SNAP-25, that even eight weeks after injection the toxin is still active and blocking vesicle-mediated release of neurotransmitter.

We found no evidence that BoNT/A protected synapses in the stratum radiatum from neurodegeneration, indeed the ratio of degenerating to intact synapses was the same in BoNT/A- and vehicle-treated animals (Fig. 5B). Absolute numbers of normal/dark terminals were also perfectly comparable between groups (Fig. 5C), ruling out an acceleration/slowing down of the degeneration process over the weeks preceding the analysis. A model in which the misfolded protein requires neural activity to kill presynaptic terminals is clearly not tenable. In the case of Aß oligomers, it has been hypothesized that their direct binding to plasma membranes of neurons could perturb the fine structure of the phospholipid bilayer [44], thus resulting in signaling changes and toxic effects proceeding via pathways largely independent of electrical activity. The lack of impact of BoNT/A on synaptic degeneration also suggests that toxic species of normal prion protein (PrP^c^) or PrP^Sc^ must initiate neurodegeneration via a receptor interaction that is not activity dependent. Similarly, our data provide little support for a sequential model, in which the disease-associated neurotoxic agent (i.e. the prion agent) first causes synaptic dysfunction and then progresses through to degeneration of the same terminals.

PrP^Sc^ may access the presynaptic element by pathways that are not dependent on vesicle exo/endocytosis. It has been shown in vitro that PrP^c^ is rapidly internalized from the cell surface and re-cycled back again in association with LRP1 [48] and the conversion of PrP^c^ to potentially toxic PrP^Sc^ may take place at the plasma membrane before being internalized by clathrin independent endocytosis [49]. If PrP^c^ also mediates the impairment of synaptic plasticity of Aß oligomers [50], [51], a similar mechanism of synaptic loss may operate in AD, although there is little evidence of degenerating synapses in experimental studies on the action of Aß peptides. It is of interest that the major effects of Aß studied to date interfere with synaptic efficacy at the post-synaptic site and inhibit long-term potentiation or enhance long-term depression [52]. Our data suggest that toxicity leading to degeneration involves different processes or perhaps different peptide from those interfering with transmission. The identification of these different species and the mechanisms of synaptic degeneration remains a significant challenge in protein misfolding diseases.
